# Consensus on a jockey’s injury prevention framework for video analysis: a modified Delphi study

**DOI:** 10.1136/bmjsem-2022-001441

**Published:** 2022-12-15

**Authors:** Daloni Lucas, Keith Stokes, Polly McGuigan, Jerry Hill, Dario Cazzola, Horse Racing Video Analysis Consensus Steering Group, Jerry Hill

**Affiliations:** 1 Department for Health, University of Bath, Bath, UK; 2 Centre for Health and Injury and Illness Prevention in Sport (CHi2PS), University of Bath, Bath, UK; 3 Centre for the Analysis of Motion, Entertainment Research & Applications (CAMERA), University of Bath, Bath, UK; 4 Medical Department, British Horseracing Authority, London, UK

**Keywords:** Consensus, Sporting injuries, Horse racing, Risk factor

## Abstract

Professional horse racing is a high-risk and dangerous sport with a high incidence of falls and injuries. While falls in horse racing are considered somewhat inevitable and carry an inherent occupational risk, little is known about the actual mechanisms of jockey injuries. Establishing injury aetiology and mechanism is a fundamental step in informing the design and implementation of future injury prevention strategies. Despite the availability of horse racing video footage, the use of video analysis to examine injury mechanisms is an underused practice. Using an expert consensus-based approach, an industry expert steering committee was assembled to develop a framework for video analysis research in horse racing. The aim of the framework is to encourage and facilitate the use of video analysis in the sport and to ensure consistency and quality of future application. To achieve consensus, a systematic review and modified Delphi method study design was used. Responses of the steering committee to two open-ended questions regarding the risk factors of falls and injury were collated and combined with findings from a literature search strategy. Appropriate descriptors and definitions were then formulated that defined and described key features of a jockey fall in horse racing and grouped into six discrete phases of an inciting event. Each member of the steering committee then examined the framework of proposed descriptors and definitions and rated their level of agreement on the 5-point Likert scale. A consensus was achieved on a total of 73 horse racing-specific descriptors and 268 associated definitions. The framework outlined in this study provides a valuable starting point for further research and practice within this area, while the recommendations and implications documented aim to facilitate the practical application of video analysis in horse racing.

## Introduction

Horse racing is a competitive, high-risk sport^1–3^ with a high incidence of falls and injuries reported.^4–10^ The most recent analysis of fall and injury rates in European horse racing was conducted by O’Connor *et al* 9 who outlined that in Irish racing, a professional jump jockey will suffer 1 fall in every 20 rides, with 20% of falls resulting in injury. In comparison, professional flat jockeys fall less frequently with 1 in every 250 rides resulting in a fall but with 35% of falls resulting in injury.

So far, injuries in horse racing have been mainly investigated from an epidemiological perspective, and while this has served to quantify the extent of injury burden faced by the sport, further research is required to understand the aetiology and mechanisms of jockey injuries. Commonly used injury prevention models^11 12^ have highlighted a need for a multidisciplinary approach to aetiological research in order to fully understand the interaction between risk factors, exposure and injury.

Studies conducted in other sports, including rugby^13 14^ and football,^15 16^ have demonstrated a reduction in injury rates following the implementation of injury prevention strategies. Fundamental to the development and design of such strategies is a thorough understanding of the inciting event. The use of video analysis is now considered commonplace in many sporting disciplines, particularly those involving collisions and a high risk of injury.^17–20^ Video analysis affords the systematic assessment of complex and dynamic sporting scenarios and is particularly useful for understanding the competitive situation, athlete behaviours and movement patterns during an inciting event.^21–23^ Such detail is often omitted from injury surveillance records. In the context of horse racing, understanding the characteristics of both injurious and non-injurious falls can directly inform the development of injury prevention strategies such as jockey education, falls technique training and safety wear development.

Despite the availability of video footage of all professional horse races, only two studies have sought to examine the kinematics involved during impact events and jockey falls.^24 25^ Perhaps such paucity of studies might be due to a lack of guidance on the use of video analysis within the horse racing setting. The absence of a sport-specific framework with clear descriptors and definitions may also hinder the application of methodologically robust video analysis and further impede the development of any injury prevention strategies that are informed by video analysis. Clearly defined descriptors and definitions improve the reliability of video analysis in a sporting context by reducing bias and subjectivity.^17 21^ Furthermore, the need for consistency and standardisation of video analysis has been previously acknowledged within other sporting disciplines and has been achieved through the development of a sport-specific analysis framework.^21 26^ Therefore, the aim of this study is to develop and achieve consensus on a framework of horse racing-specific descriptors and definitions and outline the implications, recommendations and challenges of video analysis in horse racing.

## Methods

**Figure 1:**
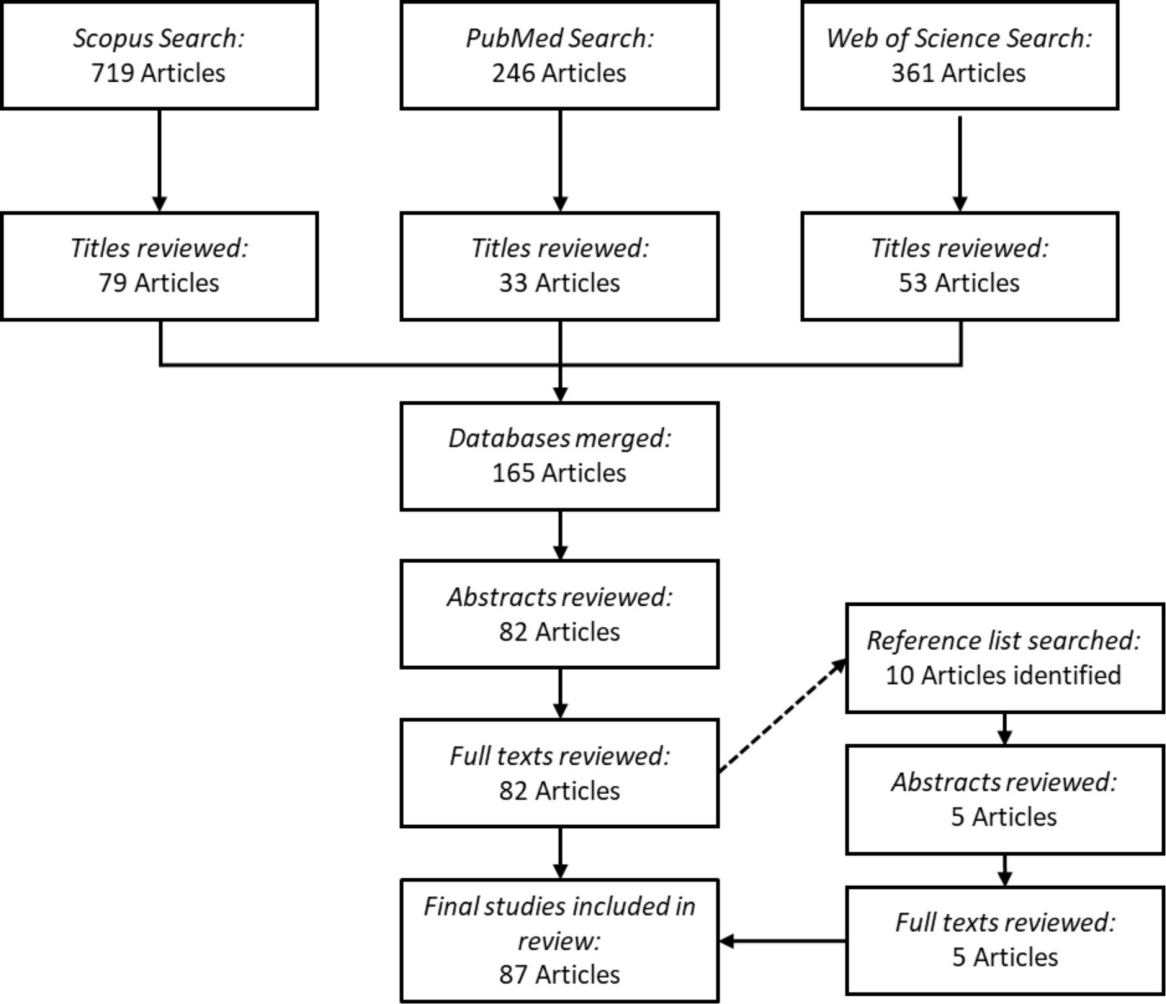
Flow diagram of literature search. Specific search terms were used to identify peer-reviewed articles in three electronic databases: PubMed, Scopus and Web of Science. The search terms were ‘horse racing’ in the title, keywords or abstract linked in any way to the following terms: ‘video analysis’, ‘jockey injury risk factors’, ‘jockey fall risk factors’, ‘jockey catastrophic injury’, ‘jockey injury mechanism’, ‘jockey injury incidence’, ‘jockey fall’, ‘jockey video analysis’ or ‘jockey injury’ anywhere in the text with a total of nine searches performed for each database. The results of all nine database searches were merged and duplicates removed. The inclusion criteria were as follows: the article needed to be published in a peer-reviewed journal in English and needed to discuss the risk factors related to jockey injuries and jockey and horse falls in horse racing. The inclusion criteria were applied at title, abstract and full-text level, any article not meeting the inclusion criteria was excluded from further review. The results from all three databases were merged and duplicates removed yielding a total of 87 articles that documented the potential risk factors of falls and jockey injuries.

To develop the framework of descriptors and definitions and reach a consensus, a two-step process was applied as previously described by Hendricks *et al* 21 with further consideration of the Conducting and REporting DElphi Studies guidelines.^27^ For the first step, a systematic search of the literature was performed. Specific search terms were used to identify peer-reviewed articles in three electronic databases: PubMed, Scopus and Web of Science. The search terms were ‘horse racing’ in the title, keywords or abstract linked in any way to the following terms: ‘video analysis’, ‘jockey injury risk factors’, ‘jockey fall risk factors’, ‘jockey catastrophic injury’, ‘jockey injury mechanism’, ‘jockey injury incidence’, ‘jockey fall’, ‘jockey video analysis’ or ‘jockey injury’ anywhere in the text with a total of nine searches performed for each database. For example, in Scopus, the full search strategy for the term ‘jockey injury’ was: (TITLE-ABS-KEY (horse AND racing) AND ALL (jockey injury) PUBYEAR<2021 LANGUAGE (English) SRCTYPE (j). If the term ‘jockey’ was omitted from the search criteria, the database yielded a heavy dominance in horse-related articles. The results of all nine database searches were merged and duplicates removed. The time frame for the literature search included any article published up to 1 November 2020. The inclusion criteria were as follows: the article needed to be published in a peer-reviewed journal in English and needed to discuss the risk factors related to jockey injuries and jockey and horse falls in horse racing. The inclusion criteria were applied at the title, abstract and full-text levels. Any article not meeting the inclusion criteria was excluded from further review. The results from all three databases were merged, and duplicates were removed, yielding a total of 87 articles that documented the potential risk factors of falls and jockey injuries. Figure 1 summarises the systematic literature search.

In step 2, the purposive recruitment of 17 steering committee members was conducted from within the British horse racing industry’s stakeholders, whose focus is on the care and well-being of professional jockeys and the industry workforce. This included representation from the jockeys’ union (Professional Jockeys Association), British horse racing’s regulatory body (British Horseracing Authority) and the jockey welfare (Injured Jockeys Fund) and training agencies (British Racing School), along with experienced medical professionals (Consultant Spinal Surgeon, Horse Racing Medical Officers) with extensive expertise in the diagnosis and management of jockey injuries. The steering committee also benefited from the representation of the British Equestrian Trade Association, which set the safety standard for body protectors used by equestrians. Finally, eight current professional jockeys (five men, three women) of varying competitive experience from both flat and jump subdisciplines also joined the steering committee.

The authorship group consisted of academics in biomechanics (DC and PM) and epidemiology (KS) and a practising physiotherapist (DL) experienced in the management and rehabilitation of jockey injuries.

Once the steering committee had been established, the development and consensus of descriptors and definitions were sought using a modified Delphi consensus method.^27–29^ The iterative process of the Delphi consensus method facilitated the development of the analysis framework while overcoming the geographical^30^ and logistical restrictions experienced due to the COVID-19 pandemic. Furthermore, this method of engagement also provided all steering committee members with the equal opportunity to contribute regardless of social status, seniority or interpersonal skills.^27 31^

A total of four Delphi rounds were conducted. For the first Delphi round, steering committee members completed an online questionnaire consisting of two open-ended questions: Q1. What do you think are the risk factors of falls in horse racing? and Q2. What do you think are the risk factors of injuries in horse racing? The authorship group then combined the risk factors proposed by the steering committee with the risk factors identified through the search strategy and drafted appropriate descriptors and definitions of such risk factors, and key features of a horse or jockey fall in horse racing.

The resultant framework of descriptors and definitions was structured into subsections to consider six discrete phases of an inciting event (ie, fall/unseat). These included the following:

- Situational descriptors—environmental conditions prior to/under which the inciting event occurred. Including location, surface type, obstacles (where relevant), competitive scenario, jockey, horse and opponent behaviour.
- Gross fall descriptors—obvious characteristics of a fall, including the type of inciting event, for example, fall or unseating.
- Flight phase descriptors—specific biomechanical characteristics of the flight/fall phase prior to jockey sustaining impact.
- Contact/impact occurrence descriptors—sequence and characteristics of impacts sustained during fall/inciting event.
- Axial skeleton descriptors—specific characteristics involving the axial skeleton during the fall/inciting event.
- Secondary impact/recovery descriptors—jockey behaviour and characteristics of any subsequent impact sustained and the recovery immediately following a fall/inciting event.

For subsequent Delphi rounds (two–four), the steering committee members convened via teleconference and examined the framework of proposed descriptors and definitions, rating their level of agreement for each on a 5-point Likert scale (1: strongly disagree; 2: disagree; 3: neither agree nor disagree; 4: agree; 5: strongly agree).^32^ An online platform (https://www.onlinesurveys.ac.uk/) was used to capture the agreement ratings and any anonymous comments or suggestions. The mean level of agreement (±95% CI) was calculated by summing the ratings and dividing this by the total number of responses. The consensus threshold was determined a priori to be 4/5 (80%). Descriptors and definitions failing to meet the consensus threshold and any anonymous comments were discussed, and any modifications or additions proposed were considered in each subsequent Delphi round. Once a consensus had been achieved for each of the descriptors and definitions, the framework composition was complete. The level of agreement for each of the six framework subsections is reported in the Results section.

To maximise objectivity and reliability and to minimise any ambiguity that might result in coding inaccuracies,^22 26 33^ three coders experienced in the analysis of human and equine movement and behaviour convened and reviewed the clarity and utilisation of the operational definitions of each analysis framework descriptor. Video footage of 20 horse racing falls (10 jump/10 flat) chosen at random from the British Horseracing Authority video archive was analysed using the video analysis framework. Coding decisions were discussed in an open forum with minimal changes deemed necessary. A single descriptor was removed from the framework due to the varying visibility of furlong markers and race start positions; therefore, coders were unable to consistently identify the distance covered prior to a fall/inciting event from the video footage alone. Furthermore, a strong preference towards coding the horse and jockey’s position in a race by grouping rather than expressed numerically was held by all analysts. While identifying position in a race is possible numerically, this requires greater time and manipulation of video footage, particularly when runners are dispersed in a race.

Finally, as not all steering committee members were available during each round, all members were offered the opportunity to review the analysis framework in its entirety and provide their definitive approval.

## Results

The search strategy yielded a total of 87 articles on risk factors related to jockey injuries and jockey and horse falls in horse racing, of which only one article provided a consensus on racing-specific definitions.^34^ Two further articles from other sporting disciplines that characterised impact events or athlete behaviour in response to an impact event were incorporated into the analysis framework.^35 36^ Following a four-round modified Delphi method, a consensus was achieved on a total of 73 horse racing-specific descriptors and 268 associated definitions that delineate the risk factors identified in the literature search strategy and Delphi round one (tables 1–6). The mean level of agreement was 4.5 (3.8–5), 4.7 (4.2–5), 4.7 (4.3–4.9), 4.7 (4.3–5), 4.8 (4.3–5) and 4.8 (4.6–5) for the situational, gross fall, flight phase, contact/impact occurrence, axial skeleton and secondary impact/recovery descriptors, respectively.

**Table 1:** Situational descriptors

Descriptor	Definition
Location of inciting event: where the jockey’s fall occurred	Parade ring^34^: the area where the horses are paraded before each race. Also referred to as the paddock
	Post-parade ring/pre-race area^34^: the area between the parade ring and the stalls/start
	Stalls/start: the box from which horses begin flat races/the collecting area behind the start line
	Racing circuit^34^: the track/area where the race is run
	Post-race to dismounting area^34^: the area between the finish line and the dismounting area
Surface type: the material of which the racetrack is made	Turf: grass
	All weather: a synthetic racing surface
Position in the race: where the horse was sitting in the race (*numerical*)	Where the horse was sitting in the race (expressed numerically)
Position in the race: where the horse was sitting in the race	Leading: front-runners
	Prominent: tracking leaders but ahead of midfield runners
	Middle division: behind leading and prominent runners but ahead of runners positioned towards the rear of the field
	Towards rear: positioned towards the back of the field
Distribution of field: the spacing between the field of horses (*subject relative to other runners*)	Grouped: horse proximity—less than a horse length apart
	Dispersed: horse proximity—more than a horse length apart
Horse performance: how the horse is immediately running prior to the inciting event (*1 s prior to fall*)	Off the bridle: horse not travelling well
	On the bridle: horse travelling well
	Pulling: horse is keen to go faster than jockey is allowing
Progression in the race: has the horse’s running position changed prior to the fall from (a) two fences prior to fall, (b) one fence prior to fall (*jump racing only*)	Yes: change in running position
	No: no changes in running position
Whip use: has the jockey used their whip prior to the inciting event? *Measured from 5 s prior to inciting event*	Yes: whip use observed
	No: no whip use observed
Whip use: has the jockey used their whip prior to the inciting event? *Measured from two fences prior (jump) or prior 30 s (flat*)	Yes: whip use observed
	No: no whip use observed
Number of whip strikes: *measured from 5 s prior to inciting event*	Count of whip strikes observed
Obstacle type: the type of obstacle at which the inciting event occurred (*jump racing only*)	Hurdle
	Plain/chase fence
	Water jump
	Open ditch
	Non-standard obstacle: cross-country/grand national fences
Location of fall—obstacle: identifies which phase of jumping the inciting event (fall/unseating) occurs	Approach: inciting event occurs during the immediate approach to the obstacle
	Take off: inciting event occurs during obstacle take off
	Flight: inciting event occurs during the flight phase of the obstacle
	Landing: inciting event occurs during obstacle landing
	Recovery: inciting event occurs during initial strides after landing
Drop fence: the landing level of the fence is lower than the take-off level	Yes: landing level lower than take-off level
	No: landing level not lower than take-off level
	Not visible/inconclusive: unable to definitively identify if the landing level is lower than the take-off level
Horse action—behavioural: any behavioural action the horse exhibits prior to the fall (*within 5 s prior to inciting event*)	Spook/shy/jink: jump sideways or a quick change of direction
	Refusal: failure of the horse to jump a fence to which he is presented. This includes any stop in forward motion
	Hesitation: horse loses forward momentum in approach to obstacle prior to take-off
	Runout: horse quickly moves sideways to go around the fence instead of jumping it, without stopping forward motion
	Bolt: running away without control
	Rear: horse stands up on its hind legs with the forelegs off the ground
	Buck/bronc: horse lowers its head and raises its hindquarters into the air while kicking out with the hind legs
Horse action—involuntary: any involuntary action the horse exhibits/experiences prior to inciting event (*within 5 s prior to inciting event*)	Stumble/trip/slip: horse loses footing while travelling on the flat
	Jump approach issue: horse experiences difficulty during the approach to the fence
	Jump take-off issue: horse experiences difficulty during take-off
	Jump flight issue: horse fails to jump cleanly and loses momentum
	Jump landing issue: horse fails to land cleanly, for example, stumble, trip, slip, peck
	Jump recovery issue: horse experiences difficulty during initial strides after landing
	Unmounted/loose horse involvement: horse’s performance impeded by a loose horse—including interference, obstruction, brought down
	Mounted horse involvement: horse’s performance impeded by a mounted horse—including interference, obstruction, brought down, clipping heels
	Other jockey involvement: horse’s performance impeded by another jockey—including interference, obstruction, brought down
	Spontaneous injury: horse sustains an injury without external influence
Jockey error: visible jockey error prior to inciting event (*within 5 s prior to inciting event*)	Failure to pull up fatigued horse
	Taking wrong course/route/obstacle
	Technical error with obstacle approach
	Clipping heels: horse contacts hind feet/legs of the horse directly in front
	Lack of control
	Loss of balance/riding position
Tack/equipment malfunction	Saddle/girth/leathers/irons
	Bridle/reins/horse’s headgear
	Horse’s shoe(s)/protective boot(s)/bandaging
	Jockey’s clothing/equipment
Jockey’s head position prior to inciting event (*immediately prior to inciting event*)	Up and forward: gaze focused on direction of travel
	Down: gaze focused on horse/track and not on direction of travel
	Motion/tracking: jockey’s gaze not fixed (head was moving)
Jockey’s body position prior to inciting event (*immediately prior to inciting event*)	Forward: jockey was in a forward 'riding position'—flexion at knees and hips
	Twisted/rotated: jockey’s trunk is rotated facing away from the direction of travel—hip and knee flexion asymmetrical
	Reclined: jockey’s trunk in a reclined position—minimal flexion at the hip
Fall warning: observed indication (stimulus) that the horse or rider might fall/become unseated, for example, horse or jockey error, injury, equipment malfunction	Yes: fall warning observed
	No: no fall warning observed
	Not visible/inconclusive: unable to definitively identify a fall warning
Attempted correction: jockey attempts to prevent the fall/unseat from occurring, for example, correct horse action, correct riding position, steer horse clear of obstruction	Yes: the jockey attempted to prevent fall from occurring
	No: the jockey did not attempt to prevent the fall from occurring
	Not possible: the jockey was unable to take any action to prevent the fall/unseat from occurring, for example, insufficient time to react, equipment damage, jockey injury
	Not visible/inconclusive: unable to definitively identify any action taken by the jockey to prevent fall/unseat from occurring

**Table 2:** Gross fall descriptors

Descriptor	Definition
Type of inciting event	Horse fall: horse’s shoulder and quarters contact the ground or the obstacle and the ground
	Jockey fall/unseated: jockey’s involuntary dismount from his/her horse
Speed of inciting event	Stationary: no visible movement
	Slow: walking
	Moderate: trot or canter
	Fast: gallop
Interaction between jockey and horse(s)	Horse fall no subsequent contact with jockey
	Horse fall—jockey contacted/trampled by own horse
	Horse fall—jockey contacted/trampled by other horse
	Horse fall—jockey contacted/trampled by own and other horse(s)
	No horse fall—jockey unseated no subsequent contact sustained
	No horse fall—jockey contacted/trampled by own horse
	No horse fall—jockey contacted/trampled by other horse(s)
	No horse fall—jockey contacted/trampled by own and other horse(s)
Number of horse fallers	Number of horses that fell during the same inciting event (count)
Number of jockey fallers	Number of jockeys that were unseated/fell during the same inciting event (count)
Jockey contact with reins (*during inciting event*)	Let go prior to landing: jockey released reins prior to landing/sustaining first impact
	Hung on to rein(s): jockey maintained contact with reins during fall and upon sustaining first impact
	Hung on to rein(s): jockey maintained contact with reins during landing/upon sustaining first impact and was dragged by horse
	Not visible
Jockey contact with stirrups (*during inciting event*)	Feet out before landing: jockey’s feet were released from the stirrup irons prior to landing/sustaining first impact
	One/both feet in irons upon landing: one/both feet remained in the stirrup irons throughout fall and during landing/sustaining first impact
	One/both feet in irons upon landing: one/both feet remained in the stirrup irons throughout fall and during landing/sustaining first impact and was dragged by horse
	Not visible
Gross outline body position (pre-impact): jockey’s body position prior to sustaining first impact	Extended: trunk and extremities extended
	Semitucked: partial flexion at trunk and/or extremities
	Tucked: both trunk and extremities flexed
Gross outline body position (during impact): jockey’s body position upon sustaining first impact	Extended: trunk and extremities extended
	Semitucked: partial flexion at trunk and/or extremities
	Tucked: both trunk and extremities flexed
Landing deceleration: did the jockey come to an immediate stop upon impact?	Yes: jockey came to immediate stop upon impact
	No: jockey did not come to an immediate stop upon impact
	Not visible/inconclusive: unable to definitively identify if jockey came to an immediate stop upon impact

**Table 3:** Flight phase descriptors

Descriptor	Definition
Flight time	Duration (ms) elapsed between jockey losing seat (which they are unable to recover) and sustaining the first contact/impact
Fall height: subjective assessment of the height at which the jockey’s fall/unseating occurs	Low: fall/unseat occurs from a height that does not exceed that of an average adult human
	Moderate: fall/unseat occurs from the height of a full-sized horse (height of the horse’s withers)
	High: fall/unseat occurs from the height of a full-sized horse in flight/jumping and exceeds that observed within the low-moderate ranges
Direction of fall: the direction by which the jockey falls from the saddle	Anterior
	Lateral
	Posterior
	Anterolateral
	Posterolateral
Flight/fall rotation (vertical axis): the amount of rotation that occurs upon the vertical axis (where observed) during the flight phase of the jockey’s fall	≤90°
	91°– 180°
	181°– 270°
	271°– 360°
Direction of rotation (vertical axis)	Clockwise
	Counterclockwise
Flight/fall rotation (mediolateral axis): the amount of rotation that occurs upon the mediolateral axis (where observed) during the flight phase of the jockey’s fall	≤90°
	91°– 180°
	181°– 270°
	271°– 360°
Direction of rotation (mediolateral axis)	Forwards
	Backwards
Flight/fall rotation (anteroposterior axis): the amount of rotation that occurs upon the anteroposterior axis (where observed) during the flight phase of the jockey’s fall	≤90°
	91°– 180°
	181°– 270°
	271°– 360°
Direction of rotation (anteroposterior axis)	Clockwise
	Counterclockwise

**Table 4:** Impact occurrence descriptors

Descriptor	Definition
Fall impact sequence: this descriptor will identify the order in which the body parts sustain impact upon 'landing'. *Code in order of occurrence and record impact intensity for each body part involved*	Head/neck
	Upper extremity (shoulder and arms)
	Torso—back
	Torso—side
	Torso—front
	Lower extremity
Site of impact: body part sustaining impact	Head/neck: above shoulder
	Shoulder: armpit to apex of shoulder level
	Arm: below armpit level
	Upper extremity: shoulder and arm
	Torso: above hip level to the level of the armpit
	Lower extremity: below hip
Impact intensity: subjective assessment of the impact sustained by the jockey	Low
	Medium
	High
Impact surface	Ground
	Obstacle—fence/railing
	Stalls
	Horse/rider
Impact surface: subjective assessment of impact surface quality	Soft: turf/sand/synthetic track/fleshy regions of the human or horse’s body
	Rigid: horse’s hoof/leg/bony prominence of horse or human/railing or equipment/concrete/obstacle (lower aspect, for example, toe board)
Movement speed of impact surface: the speed at which the impact surface was travelling during the point of contact	Stationary
	Slow
	Moderate
	Fast
Movement direction of contact surface: the direction the impact surface was travelling at the point of contact with the jockey	Towards jockey
	Away from jockey
	Not applicable (stationary)
	Not visible

**Table 5:** Axial skeleton descriptors

Descriptor	Definition
Head and neck position during *high* impact: identifies the alignment of the head and neck during any impact that has been identified as *HIGH (*see table 4 *)* or *has occurred to the head*. Where multiple high impacts have been sustained, code head and neck alignment for each high impact sustained. *Combined movement (code each discrete movement observed*)	Neutral
	Flexion
	Extension
	Lateral flexion—right
	Lateral flexion—left
	Rotation—right
	Rotation—left
Torso position during *high* impact: identifies the alignment of the torso during any impact that has been identified as *HIGH (*see table 4 *)* or *has occurred to the head*. Where multiple high impacts have been sustained, code torso alignment for each high impact sustained. *Combined movement (code each discrete movement observed*)	Neutral
	Flexion
	Extension
	Lateral flexion—right
	Lateral flexion—left
	Rotation—right
	Rotation—left
Axial skeleton orientation upon *commencement* of fall/unseat: the orientation of the head in relation to the pelvis upon commencement of fall/unseat. *Please note: the commencement of a fall as previously defined is the point at which the jockey parts company from his/her horse from which they cannot recover. Reference points/landmarks: 1. head—apex of helmet; 2. pelvis—top of the breeches (as this can be easily distinguished from the bottom of the silks*)	Head above the height of the pelvis
	Head level with pelvis
	Head below the height of the pelvis
Axial skeleton orientation upon *impact*: the orientation of the head in relation to the pelvis during *initial* impact. *Reference points/landmarks: 1. head—apex of helmet; 2. pelvis—top of the breeches (as this can be easily distinguished from the bottom of the silks*)	Head above the height of the pelvis
	Head level with pelvis
	Head below the height of the pelvis
Angle of impact: observed angle between the longitudinal axis of the identified body part and the impact surface upon impact	Vertical
	Angled
Head impact sustained: identifies if jockey sustained an impact to the head during inciting event	Head impact sustained
	No head impact sustained
	Not visible
Head impact location: identifies the anatomical region of the head where the impact is sustained, *for example, forehead=anterior*	Anterior
	Posterior
	Lateral—right
	Lateral—left
	Anterolateral—right
	Anterolateral—left
	Posterolateral—right
	Posterolateral—left
Head impact intensity: subjective description of the impact sustained by the jockey’s head	Low
	Moderate
	High
Head impact surface: the surface which the jockey’s head impacts	Ground
	Obstacle—fence/railing
	Stalls
	Horse/rider
Head movement direction: identifies biomechanical plane(s) of the jockey’s head motion upon impact	Sagittal: forward–backward movements^36^
	Coronal: side-to-side movements^36^
	Transverse: rotational or twisting movements^36^
	Multiplane: movements incorporating more than one plane^36^
	Not visible/inconclusive: unable to definitively identify plane^36^
Head rebound: identifies whether the jockey’s head rebounds immediately after impact	Yes: head rebound visible
	No: no head rebound visible
	Not visible/inconclusive: unable to definitively identify any head rebound
Head acceleration: identifies biomechanical plane(s) of jockey’s head motion (where acceleration observed, and no impact sustained)	Sagittal: forward–backward movements^36^
	Coronal: side-to-side movements^36^
	Transverse: rotational or twisting movements^36^
	Multiplane: movements incorporating more than one plane^36^
	Not visible/inconclusive: unable to definitively identify plane^36^
Observed signs of possible concussion^35^	Lying motionless: lying without purposeful movement on the racetrack, for >2 s*. Does not appear to move or react purposefully, respond or reply appropriately to the race situation.^35^
	Motor incoordination: appears unsteady on feet (including losing balance, staggering/stumbling, struggling to get up, falling) or in the upper limbs (including fumbling). May occur in rising from the racetrack surface or in the motion of walking/running/skating^35^
	Impact seizure: involuntary clonic movements that comprise periods of asymmetric and irregular rhythmic jerking of axial or limb muscles^35^
	Tonic posturing: involuntary sustained contraction of one or more limbs (typically upper limbs), so that the limb is held stiff despite the influence of gravity or the position of the jockey. The tonic posturing could involve other muscles, such as the cervical, axial and lower limb muscles. Tonic posturing may be observed while the jockey is on the racetrack surface or in the motion of falling, where the jockey may also demonstrate no protective action* (*this was previously known as no protective action—stiff)^35^
	No protective action—floppy: falls to the playing surface in an unprotected manner (ie, without stretching out hands or arms to lessen or minimise the fall) after direct or indirect contact with the head. The jockey demonstrates a loss of motor tone (which may be observed in the limbs and/or neck) before landing on the racetrack surface^35^
	Blank/vacant look: the jockey exhibits no facial expression or apparent emotion in response to the environment* (*may include a lack of focus/attention of vision; blank/vacant look is best appreciated in reference to the jockey’s normal or expected facial expression)^35^

*>2s for removal and assessment of the jockey. Significantly longer periods of lying motionless may necessitate immediate and permanent removal from play, depending on the circumstances

**Table 6:** Subsequent impact descriptors

Descriptor	Definition
Did the jockey actively tuck and roll: tuck and roll=jockey adopts fetal position then rolls across the ground *(this is considered a protective posture whereby the jockey reduces their physical outline)*	Yes: the jockey demonstrated an active tuck and roll
	No: the jockey did not demonstrate an active tuck and roll
	Not visible/inconclusive: unable to definitively identify jockey action
If YES, when did jockey commence tuck? *Tuck=adopt fetal position/flex to reduce bodily outline*	Prior to landing: jockey adopted tucked position before initial impact
	Upon landing: jockey tucked upon sustaining initial impact
	After landing: jockey tucked while lying on ground (delayed)
	Not visible/inconclusive: unable to definitively identify any jockey action
Number of times the jockey rolls while on the ground	Count of jockey 'rolls' (complete revolution)
Did jockey sustain any subsequent (secondary/tertiary) impact(s)? Identifies if the jockey sustained any further impact(s) during the inciting event	Yes: jockey sustained subsequent impact(s)
	No: jockey did not sustain any subsequent impact(s)
	Not visible/inconclusive: unable to definitively identify any subsequent impact
Subsequent impact description	Brief strike or glancing blow: kick/trample/stomp
	Prolonged compression: crush
	Not visible/inconclusive: unable to definitively identify type of secondary impact
Number of subsequent impact(s)	Single: jockey sustained a single secondary impact
	Multiple: jockey sustained multiple impacts
Site of impact: body part(s) sustaining impact	Multiple body sites
	Head/neck: above shoulder
	Shoulder: armpit to apex of shoulder level
	Arm: below armpit level
	Upper extremity: shoulder and arm
	Torso: above hip level to the level of the armpit
	Lower extremity: below hip
Impact intensity: subjective assessment of the impact sustained by the jockey	Low
	Medium
	High
Movement speed of contact surface: the speed at which the contact surface was travelling during the point of impact	Stationary
	Slow
	Moderate
	Fast
Movement direction of contact surface: the direction the contact surface was travelling at the point of impact with the jockey	Towards jockey: contact surface moved towards jockey
	Away from jockey: contact surface moved away from jockey
	Not applicable: stationary/static surface
Did the jockey attempt to avoid any subsequent impact?	Yes: jockey attempted to avoid any subsequent impact
	No: jockey did not attempt to avoid any subsequent impact
	Not visible/inconclusive: unable to definitively jockey action
Jockey recovery following the fall: what action did the jockey take on completion of the fall? (*during the 10 s immediately following inciting event*)	Stood following fall
	Attempted to stand following the fall
	Crawled on hands and knees
	Sat following fall
	Remained on ground
Duration on the ground following fall (*seconds*)	Duration: jockey remains on the ground after fall
	Not visible/inconclusive: unable to quantify duration on ground

### Dissenting viewpoints

The decision to include descriptors that capture falls either prior to or immediately after the race generated much debate within the steering committee, with these failing to meet the consensus threshold. It was deemed that pre-race and post-race inciting events were not relevant for inclusion by 4 of the 15 steering committee members in attendance during round two. However, a limitation cited within a previous epidemiological study is due to the reporting of fall characteristics only for the duration of a race. This accounts for a proportion of the time the jockey is mounted on the horse.^5^ Pre-race activities accounted for 47% of jockey falls, while post-race activity accounted for 11% of jockey falls in flat racing.^37^ The mechanisms of such injuries are not fully understood, with the suggestion of jockey fatigue being associated with post-race falls,^5^ confirmation of this suspicion may indicate the need for improvements in jockey fitness as a fall mitigation strategy. A potential barrier to performing video analysis of the horse and jockey interaction outside of the race scenario may be due to the lack of camera coverage or preserved video footage capturing this duration.

## Discussion

The aim of this study was to develop and achieve consensus on a framework of sports-specific descriptors and definitions to facilitate the systematic use of video analysis in horse racing. The current study identified a total of 73 horse racing-specific descriptors and 268 associated definitions, which were organised into subsections to consider six discrete phases of an inciting event.

### Injury prevention through jockey education and training

Descriptors that capture the action of the horse be this behavioural, for example, refusal or involutory, for example, spontaneous injury, were included within the analysis framework. Hitchens *et al* 38 identified that jockeys were 171 times more likely to be injured if their horse sustained a catastrophic injury which resulted in a fall. A relationship between whip use and horse falls has also been identified^39^; horses that were being whipped and progressing through the race were at greater risk of falling compared with horses that had no whip encouragement. An increase in whip use may be due to equine fatigue or poor performance due to discomfort associated with an impending injury.^39–41^ Professional jockeys are therefore required to recognise signs of equine fatigue or distress and, in doing so, ensure the elective removal from a race, further mitigating the risk of a fall^41^ and subsequent injury. Video analysis capturing such events may prove a useful tool in jockey education, and descriptors to capture these risk factors are included within the framework.

Examining the behaviours of jockeys and characteristics associated with injurious and non-injurious falls will aid the identification of high-risk scenarios to be avoided, for example, maintaining contact with the reins or stirrup irons during a fall. Conversely, protective actions may also be identified, such as reducing the bodily outline upon landing, which may decrease the risk of sustaining any subsequent impact from kicks while on the ground. Quantifying these behaviours and actions will inform the content and delivery of fall training programmes. Such training exists but is based purely on anecdotal observations.

### Clinical implications

The infrastructure to facilitate the real-time analysis of video footage already exists as stewards and judges observe for any rule infringements such as improper whip use and accidental interference.^42^ This real-time analysis could also be extended to aid the detection and management of injuries sustained through falls. The use of video analysis has been successfully implemented in professional rugby unions to assist in the identification of sport-related concussions.^18 35^ Despite the under-reporting of concussive events in horse racing injury surveillance records,^43^ concussion is the most frequent head injury endured by jockeys (jump 47.5; flat 10.8 per 1000 race meetings).^44^ The introduction of mandatory medical assessment has sought to deter under-reporting.^43^ However, the addition of video analysis for the identification of signs of concussion could strengthen existing assessment protocols further. Furthermore, the inclusion of the descriptors and definitions outlined by Davis *et al* 35 in the international consensus definitions of video signs in professional sport may facilitate the identification of visible sports-related concussions in horse racing.

### Integration of video analysis with additional data sources

In the absence of microtechnology worn by the jockey, such as global positioning system and accelerometry, the speed of an inciting event and the impact intensity sustained can be subjectively estimated using the framework descriptors outlined. Such approaches are used effectively in the video analysis of rugby union and league tackles.^19 21 36^ However, the accuracy and reliability of these subjective measures within horse racing are yet to be determined. Future integration of video analysis with objective data sources that quantify variables such as velocity and impact force will improve our understanding of exposure and the aetiology of horse racing injuries.

Where possible, descriptors and definitions outlined in the European consensus on epidemiological studies of injuries in the thoroughbred horse racing industry^34^ have been incorporated into the framework. This is intended to strengthen the future convergence of video analysis and injury surveillance data and facilitate consistency in the reporting of injuries. Certain detail regarding intrinsic (eg, injury history, bone health) and extrinsic (eg, going) risk factors which may predispose an individual, potentially making them susceptible to injury,^45–47^ is not attainable from video analysis alone.^21^ Equally, injury surveillance data alone do not provide enough detail regarding the inciting event to design and develop injury prevention strategies, especially if jockey behaviour is the target of an intervention.^21 46^ Therefore, video analysis should be integrated with injury surveillance data to facilitate a multifactorial approach to injury prevention in horse racing.

### Practical challenges/recommendations

Ultimately, the quality of available video footage may have a direct impact on the extent of analysis possible. Horse racing video footage is typically captured by cameras mounted on moving vehicles that pursue the field of runners as they progress through the race. The varying racecourse topographies can present a challenge in accessing a suitable camera angle and may result in vital moments being obscured from view. Furthermore, uncalibrated video footage will limit the possibility for a more quantitative biomechanical analysis of injury mechanisms and is, therefore, largely dependent on the categorisation and subjective assessment of observed actions. Model-based image-matching techniques have been used to extract human motion from uncalibrated video footage. However, its accuracy and validity are dependent on the availability of multiple views and camera angles.^48^ The descriptors and definitions within this framework set to identify and describe the gross movement patterns involved in jockey falls, while an accurate analysis of the external and internal biomechanical loads experienced during a fall is only possible during controlled experimental conditions (eg, laboratory studies of staged falls) due to the safety implications and the practicalities of such investigation in horse racing.

The acquisition of racecourse maps may provide orientation and awareness of environmental characteristics (eg, position of obstacles, severity of bend) of an inciting event, thus providing context to the video footage being analysed. Furthermore, racecourse maps may be electronically integrated with specialist video analysis software which will allow for the location of an inciting event to be plotted directly onto the map allowing for improved data visualisation.

**Figure 2:**
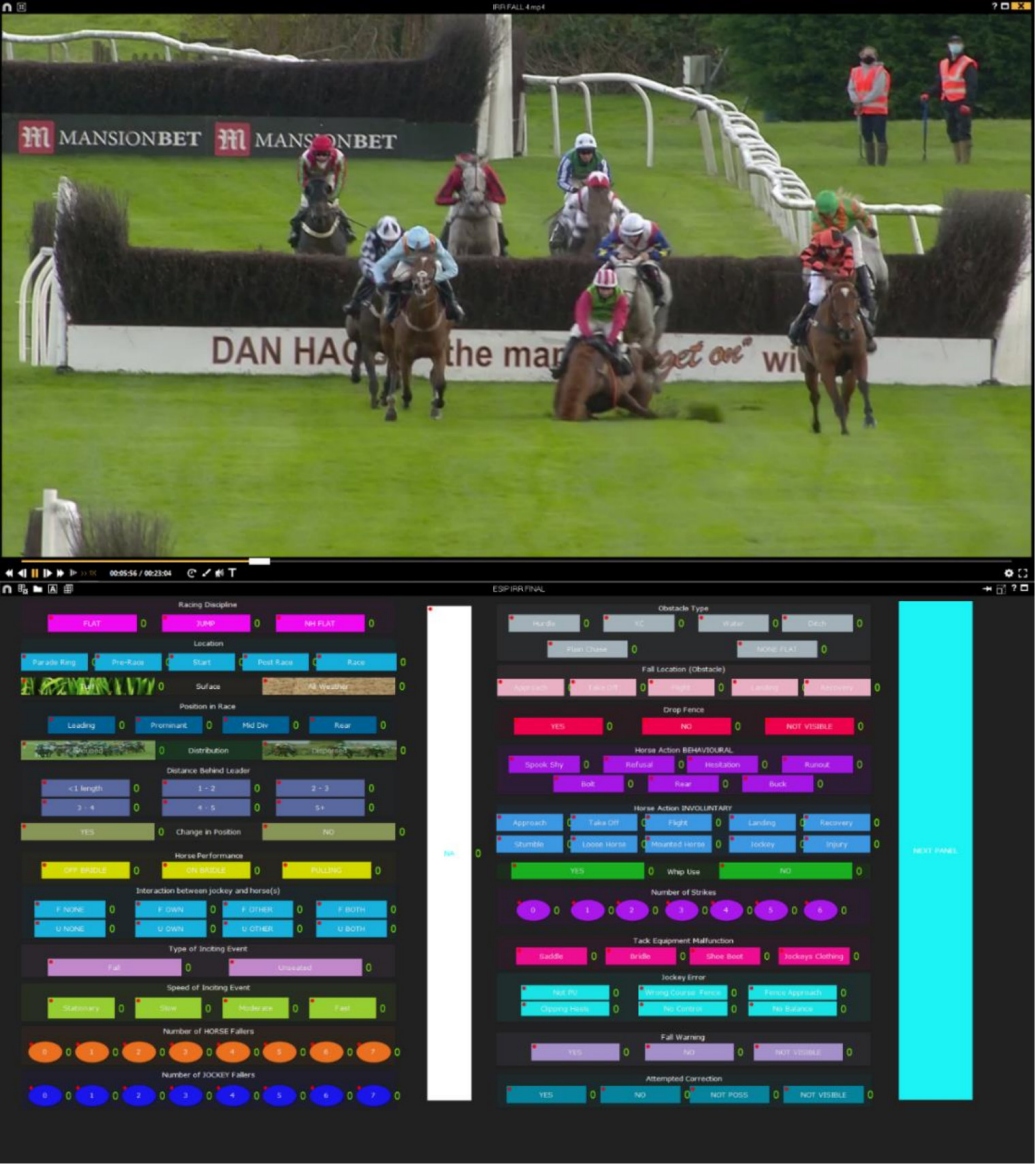
Example of video analysis coding window using Nacsport Pro Plus software. Specialist video analysis software allows the video to be manipulated and analysed (top) while the appropriate descriptor can be simultaneously selected by clicking on the relevant button within the custom coding window (bottom).

Although not essential, specialist video analysis software may facilitate and expedite the coding process, particularly when the number of variables being considered is vast. Certain software programs feature utilities to capture, tag, compare and annotate video footage. Figure 2 shows an example of software coding window (Nacsport, Spain) which allows the video to be analysed and the appropriate descriptor and definition to be simultaneously selected by clicking on the relevant button, thus, improving coding efficiency and allowing for subsequent quantitative analysis of the recorded actions. However, the use of specialist software programs carries an expense and is dependent on technical knowledge and capability. As a minimum requirement, the software used to navigate and view video footage should allow for frame-by-frame analysis and slow-motion playback speeds.

### Reliability procedures

In video analysis, reliability is a measure of consistency between ratings made by either the same rater (intrarater reliability) or between two different raters (inter-rater reliability).^49^ The method used to examine reliability will be dependent on the type of variable (continuous vs categorical) being considered, for example, number of whip strikes versus location of fall.^33^ The use of video analysis to observe postural alignment and quantify joint angles is challenging, with the reliability differing between descriptors.^26^ The operational definitions outlined within the horse racing video analysis framework look to improve coding accuracy and consistency.

Reliability testing during a video analysis project should be considered a process.^21^ Repeating reliability measures at the beginning and at later phases of data collection will identify any analysis issues that need to be addressed, therefore facilitating acceptable intrarater and inter-rater reliability, and improving the quality of any project output.^21 22 26 33^

### Limitations

The availability of quality video footage within amateur racing is currently somewhat lacking. Therefore, only footage featuring professional horse racing was used in the development of the video analysis framework. Consequentially, the application of the framework for the analysis of amateur racing will require further validation. Similarly, the terminology featured within the framework is specific to that of British horse racing. The application of the framework by international racing jurisdictions will need to be reviewed to accommodate the potential differences in country-specific horse racing terminology and rules.

A further limitation to be acknowledged is that not all members of the steering committee could participate in each of the four Delphi rounds. A lack of individual availability prevented full attendance; it was, however, deemed imperative that adequate representation of the professional jockeys was present in order to proceed. To further mitigate the impact of steering committee unavailability, all members were offered the opportunity to consider the final framework in its entirety and provide their approval.

## Conclusion

The aim of this study was to develop and achieve a consensus on a framework of sports-specific descriptors and definitions to facilitate the systematic use of video analysis in horse racing. The framework outlined provides a valuable starting point for further research and practice. Which variables to use will be dependent on the objectives of future study. While falls in horse racing are considered somewhat inevitable and carry an inherent occupational risk, understanding the complex dynamics at play is imperative for jockey and equine welfare.

10.1136/bmjsem-2022-001441.supp1 Supplementary data
