# Supplementary material for: Consensus on a jockey’s injury prevention framework for video analysis: a modified Delphi study
Source: BMJ Open Sport Exerc Med. 2022 Dec 15;8(4):e001441. doi: 10.1136/bmjsem-2022-001441 (PMC9756174; doi:10.1136/bmjsem-2022-001441)
Supplement: Supplementary data [file bmjsem-2022-001441supp001.pdf]

Supplementary Material

Consensus on a jockey's injury prevention framework for video analysis: a modified Delphi study

Contents

Supplementary Material 1

Consensus on a jockey's injury prevention framework for video analysis: a modified Delphi study. 1

Appendix 1: 2

Horse Racing Video Analysis Consensus Group 2

Appendix 2: 3

Figure 1: Flow chart of Delphi process 3

Appendix 3: 4

Table 1: Table showing analysis phase description 4

Appendix 4: 5

Table 2: Dates and number of attendees per Delphi round 5

Appendix 5: 6

Table 3: Number of descriptors and mean level of agreement for each analysis phase 6

Lucas D, *et al. BMJ Open Sp Ex Med* 2022; 8:e001441. doi: 10.1136/bmjsem-2022-001441

Appendix 1:

Horse Racing Video Analysis Consensus Group

1. Dr Jerry Hill - Chief Medical Advisor, British Horseracing Authority, London, England
2. Mr Richard Perham - Senior Jockey Coach, British Racing School, Newmarket, England
3. Mrs Claire Williams - Executive Director, British Equestrian Trade Association, Wetherby, England
4. Ms Lisa Hancock - Chief Executive, The Injured Jockeys Fund, Newmarket, England
5. Dr Anna Louise McKinnon - Head of Clinical Services, The Injured Jockeys Fund, Newmarket, England
6. Mr Paul Struthers - Former Chief Executive, The Professional Jockeys Association, Newbury, England
7. Mr Jason Harvey - Consultant Spinal Surgeon, Fortius Clinic, London, England
8. Prof. Michael Gilchrist - Medical Engineering, University College Dublin, Dublin, Ireland
9. Ms Kerry Kuznik - Medical Assistant and Jockey Athlete Research Coordinator, British Horseracing Authority, London, England
10. Mr Kevin Jones - Professional Jump Jockey, England
11. Miss Page Fuller - Professional Jump Jockey, England
12. Mr Jamie Moore - Professional Jump Jockey, England
13. Miss Bryony Frost - Professional Jump Jockey, England
14. Ms Hollie Doyle - Professional Flat Jockey, England
15. Mr Jim Crowley - Professional Flat Jockey, England
16. Mr David Egan - Professional Flat Jockey, England
17. Mr Tom Marquand - Professional Flat Jockey, England

Appendix 2:

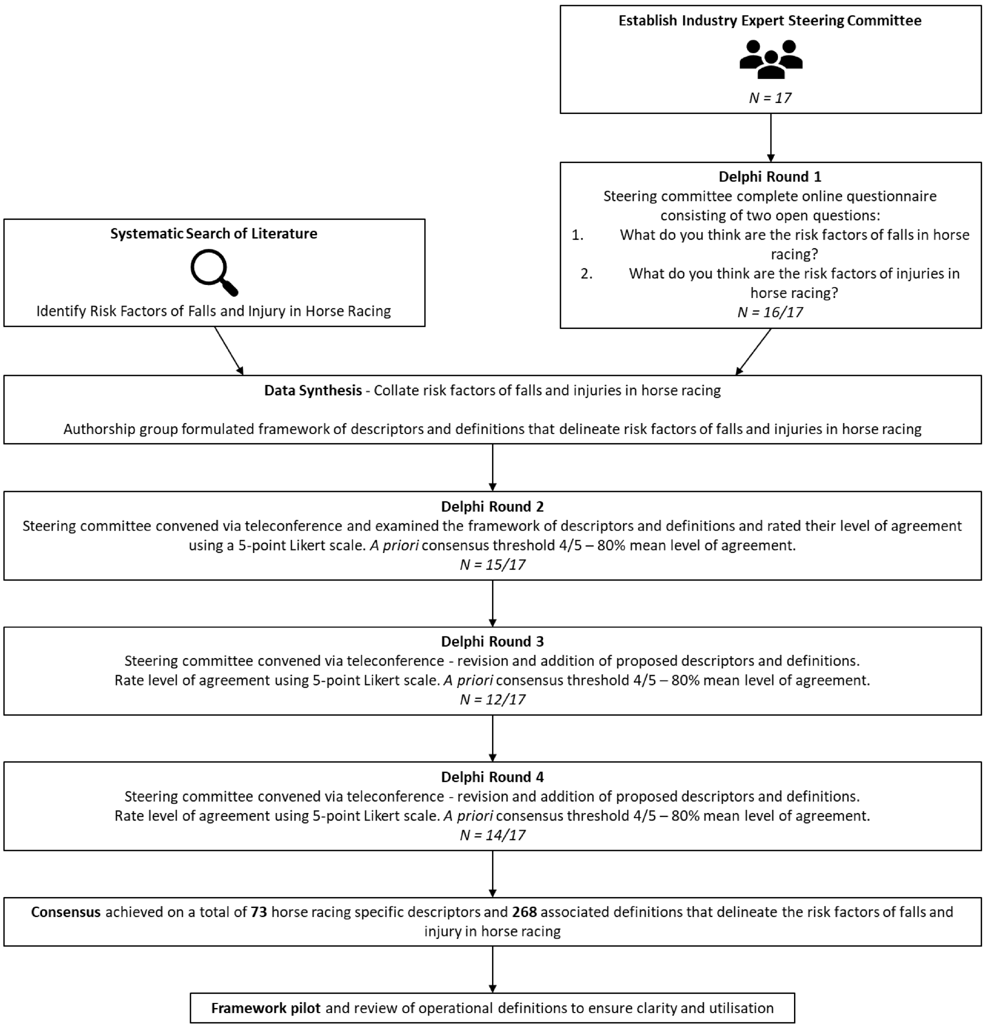

Figure 1: Flow chart of Delphi process

Appendix 3:

Table 1: Table showing analysis phase description

Analysis Phase	Analysis Phase Description
**Situational**	Environmental conditions prior to / under which the inciting event occurred. Including, location, surface type, obstacles (where relevant), competitive scenario, jockey, horse and opponent behaviour
**Gross Fall**	Obvious characteristics of fall including type of inciting event e.g., fall or unseating
**Flight Phase**	Specific biomechanical characteristics of the flight / fall phase prior to jockey sustaining impact
**Contact Occurrence**	Sequence and characteristics of impacts sustained during fall / inciting event
**Axial Skeleton**	Specific characteristics involving the axial skeleton during the fall / inciting event
**Subsequent Impact / Recovery**	Jockey behaviour and characteristics of any subsequent impact sustained and the recovery immediately following a fall / inciting event

Lucas D, *et al. BMJ Open Sp Ex Med* 2022; 8:e001441. doi: 10.1136/bmjsem-2022-001441

Appendix 4:

Table 2: Dates and number of attendees per Delphi round

Delphi Round	Date	Number Invited	Number of Attendees
**1**	25/09/2020	17	16
**2**	05/11/2020	17	15
**3**	17/12/2020	17	12
**4**	02/02/2021	17	14

Lucas D, *et al. BMJ Open Sp Ex Med* 2022; 8:e001441. doi: 10.1136/bmjsem-2022-001441

Appendix 5:

Table 3: Number of descriptors and mean level of agreement for each analysis phase

Analysis Phase / Subsection	Number of Descriptors	Number of Definitions	Mean Level of Agreement
**Situational**	21	77	4.5 (3.8 – 5)
**Gross Fall**	10	33	4.7 (4.2 – 5)
**Flight Phase**	9	27	4.7 (4.3 – 4.9)
**Contact Occurrence**	7	29	4.7 (4.3 – 5)
**Axial Skeleton**	13	59	4.8 (4.3 – 5)
**Subsequent Impact**	13	43	4.8 (4.6 – 5)

Lucas D, *et al. BMJ Open Sp Ex Med* 2022; 8:e001441. doi: 10.1136/bmjsem-2022-001441
